# A specific QSAR model for proteasome inhibitors from Oleaeuropaea and Ficuscarica

**DOI:** 10.6026/97320630014384

**Published:** 2018-07-31

**Authors:** Lahmadi Ayoub, El-aliani Aissam, Kasmi Yassine, Elantri Said, El Mzibri Mohammed, Aboudkhil Souad

**Affiliations:** 1 Laboratory of Biochemistry, Environment and Agri-Food (URAC 36)-Faculty of sciences and techniques - Mohammedia, Hassan II university Casablanca Morocco; 2 Unit of Biology and Medical Research, National Center for Energy, Nuclear Science and Technology. Morocco; 3 Green Biotechnology Team, Moroccan Foundation for Advanced Science, Innovation and Research (MAScIR), Mohammadia School of Engineering, Rabat Design Center, Mohammed V University, Morocco

**Keywords:** Proteasome, inhibitors, QSAR, *Oleaeuropaea*, *Ficuscarica*

## Abstract

*Oleaeuropaea* and *Ficuscarica* are widely used in traditional medicine for the treatment of cancer. Therefore, it is of interest to develop a QSAR model for screening proteasome inhibitors from plant source. Hence, a QSAR model was developed using multiple linear regressions; partial least squares regression and principal component regression methods. Results of QSAR modeling and docking demonstrate that compounds derived from both plants have great potentiality to be proteasome inhibitors. The developed QSAR model highlights a strong structure-effect relationship. The predicted correlation of comparative molecular field analysis, and comparative molecular similarity indexes are 0.963 and 0.919, respectively. Computed absorption, distribution, metabolism, excretion and toxicity studies on these derivatives showed encouraging results with very low toxicity, distribution and absorption.

## Background

**Figure 1:**
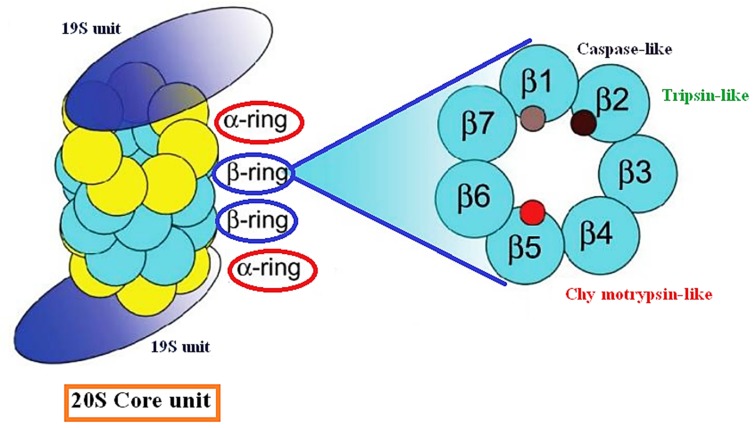
Schematic representation of the proteasome 26 S structure.20S: core particle of Two alpha rings and two beta rings in which reside all catalytic activities (β1: Caspase-like; β2: Tripsin-like and β3: Chymotrypsin-like); 19S: regulator unit

Ubiquitin-Proteasome System (UPS) is considered as a multisubunits protease complex playing a crucial role in the maintenance of cellular homeostasis by the degradation of more than 80% of poly-ubiquitinate dcytosolic proteins, including proteins involved in the regulation of the cell cycle, cell differentiation, immune defense, stress response and programmed cell death [1, 2]. The UPS is a highly organized structure composed of one central barrel-shaped catalytic core particle 20S, comprising four stacked, seven membered rings, two outer α and two inner β rings (Figure 1) [3, 4]. The beta-type subunits are more variable and appear to have a catalytic activity such as caspase-like (PGPH), trypsin-like (T-like) and chymotrypsin-like (ChT-like) activities leading proteins degradation [3, 4]. In contrast, alpha subunit is structural in natureand serves as a docking domain for the regulatory particles and forms a gate that blocks unregulated access of substrates to the interior cavityof the complex [5]. Several studies have demonstrated that the dysregulation of the proteasome has been involved in neurodegenerative diseases and cancer like multiple myeloma [6], hepatocellular carcinoma [7] and melanoma [8], making the ubiquitin-proteasome system as one of the most promising targets in cancer therapy [1]. Therefore, many studies have proved that proteasome is an anticancer target validated by remarkable clinical successes of proteasome inhibitor drugs such as bortezomib, carfilzomib and ixazomib [9]. However, the potential side effect by extended treatment is evident. Hence, it is of interest to use plant materials to identify proteasome inhibitors [10].

Oleo europaea L. and Ficuscarica L. are widely used in traditional medicine to treat metabolic, respiratory, cardiovascular, antispasmodic, anti-inflammatory, eyesore and cancer diseases [11, 12, 13]. Furthermore, previous studies have demonstrated the ability of these plants' extracts to inhibit the proliferation of several cancer cell lines including pancreatic [14], leukemia [15], stomach [16], breast [17, 18], prostate [19] and colorectal cancer [20]. Several studies have reported that pharmacological properties of Ficuscarica L. and Oleaeuropaea L. are probably due to the presence of plant secondary metabolites, prevailing in several bioactive compounds, such as polyphenols, flavonoids, tannins, organic acids, coumarins, vitamin E and carotenoids [21, 22, 23, 24]. These metabolites are well documented and all studies converge to their antioxidant power preventing a wide range of degenerative diseases [25]. Therefore, it is of interest to develop QSAR models for proteasome

## Methodology

### Chemical compound

Plants compounds were collected from the PubChem database. A total of 71 components, reported to be isolated from Ficuscarica L. (31 compounds) and Oleaeuropaea L. (40 compounds) were selected for this study. The list of these molecules and their accession number are reported as supplementary data. Moreover, 30 compounds, used in chemotherapy targeting the proteasome chymotrypsin-like activity, were also included in this study. Among them, 19 compounds were used to set-up the model (Supplementary Data) and the 11 remaining compounds were used for its validation (Supplementary Data - available with authors).

### QSAR 2D and 3D

Quantitative structure - activity relationship (QSAR) was established using the MEO software version 8 and XLSTAT version 2016. In this assay, the activity was evaluated using the IC50 of Chymotrypsin-like activity of Proteasome. The model was established by PSL and PCR methods.

All non - significant descriptors, which they have values equal 0 or having the same values for all the molecules are removed automatically. Moreover, the descriptors that have a correlation more than 75% are also eliminated. PCA was used to ease the pool of calculated structural descriptors and thus used to help decide on a suitable model more difficult for further analysis.

To obtain the equation of correlation, gradient boosting procedure was used. It is widely accepted that among all methods used in QSAR, RFs and Stochastic Gradient Boosting (SGB) are the best performers' methods [26]. Moreover, some pharmacodynamics and kinetics descriptors, including the number of aromatics, number (NB) of Carbon, NB of hydrogen and type of bonds, were also used.

The optimum number of components giving less root mean square deviation (RMSD) of prediction and high regression (r2) were retained. In addition, the regression (r2), number of components, the conventional correlation coefficient (r2) and its RMSD were also computed for model.

The test set was extracted from the homogenized calibration set. For the present work, the selection of the test set was carried out on the basis of the hierarchical grouping technique.

The models obtained were validated by the Y-Randomization method. The dependent vector is mixed randomly several times. A new QSAR model is developed after iteration. New QSAR models should have lower Q2 and R2 values than the original models. This technique is done to eliminate the possibility of chance correlation. If higher values of q2 and r2 are obtained, this means that an acceptable QSAR cannot be generated for this dataset due to structural redundancy and chance correlation.

### Docking

Interactions between ligands and the proteasome 20S (download from RCSB Database with the code 4R3O), were evaluated using Autodock software. The results were visualized using Chimera and PyMol software [5].

### ADMET proprieties

Pharmacokinetics is a drug discovery process that describes the totality of all parameters of drug circulation in the body. ADMET profile evaluation is widely used to evaluate the potential pharmacokinetic characteristics of chemical compounds. These parameters include the absorption of the drug (absorption), the distribution in the body (distribution), the biochemical remodeling (metabolism) and the excretion. In this study, ADMET analysis was performed using Pre - ADMET server and ADMET-Sar [27, 28].

## Results & Discussion

### QSAR analysis

**Figure 2:**
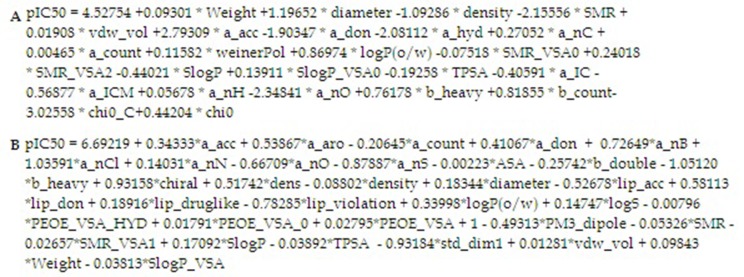
Generated 2D (A) and 3D (B) equations for IC50 prediction; Smr : Molecular refractivity ; Vdm :_vol vend derwaal volume ; a_acc : nb H-bond acceptro atoms ; a_don : nb H-bond donor atoms ; a_hydro : bond hydrogenic ; nC : nb carbon ; a_count : nb of atoms ; weinerPol : weiner polarity number ; logP(o/w) : log octanal/water partition coeffeession ; SMR vsa0 : Bin0 SMR ; SlogP : Log Octonal water parition ; TPSA topological polar surface ; ICM : aromatic information content ; nO : nboxyden ; heavy : nb heavy atoms ; Chi : chric carbon.

**Figure 3:**
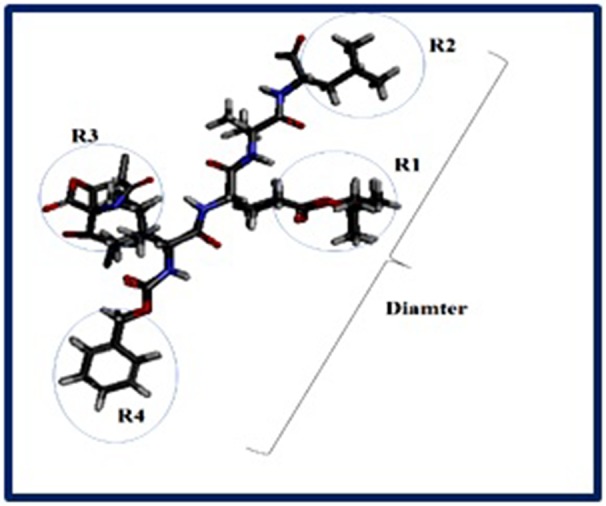
Schematic representation of the main core found in most proteasome inhibitors used as reference drugs with their major radicals; Rx is a serie of hydrophobic bonds. Aromatic nucleus 5, 6 or 7 with or without N. R2 R are a [CH] - O - features or aromatics. However, F, O, or N can change the C4 C5and the R3 can be aromatics with link to phosphate in position C6.

**Figure 4:**
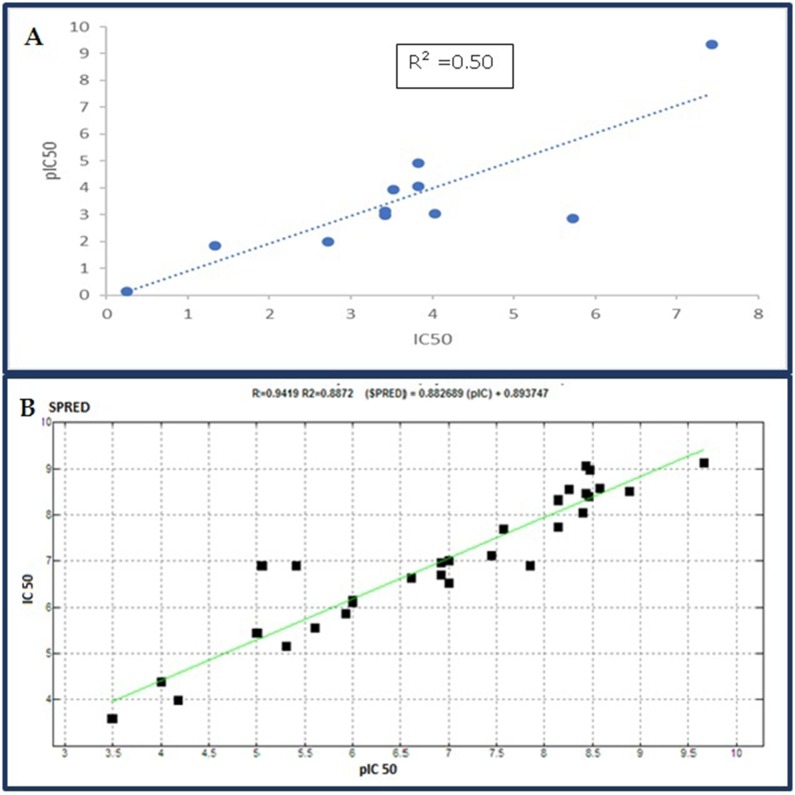
Validation of developed models; A: correlation between predicted and real IC50 for 2D model validation. B: correlation between predicted and real IC50 for 3D model validation.

In this study, we have used referential drugs widely used as inhibitors of proteasome and targeting the Chymotrypsin-like activity, to generate 2Dand 3D models. Firstly, we have generated the 2D model using the real IC50 of 19 reference drugs (supplementary data) and calculated the diameter and Lipinski parameters of these drugs. The generated 2D model is reported in Figure 2 and the principal structural radicals are illustrated in Figure 3. Validation of this model was done using the remaining 11 reference drugs by comparing the diameter and Lipinski parameters with the in vitro IC50 reported for these drugs. The correlation between the predictive IC50 and the pharmacokinetics values, assessed by the real IC50, is reported in Figure 4 and highlights a correlation of approximately 70% (r2: 0.89).

Moreover, results showed that the real IC50 of the reference drugs is proportional to the diameter of the drug. This can be explained by the presence of long active sites that could be targeted by tested ligands as well as the nature and structure of proteasome.

Using 3D parameters, we have generated a 3D model containing 34 parameters, including number of oxygen, number of carbons, number of the aromatics and, the energy and diameter as well as the Lipinski parameters (Figure 2 ). Using this model, comparison between predicted and observed activities showed high correlation with r2 of 0.98. The stable conformation of the 3D structure is very important to develop reliable and repetitive 3D - QSAR models. In this study, MOE was used to search for lowest energy 3D conformations and the PLS analysis was used to construct a linear correlation between the subset of descriptors and the bioactivities. To select the best model, the cross - validation was performed to reduce the square of cross validation coefficient (q2) and the optimum number of principal components. Difference between r2 and q2 should not be more than 0.3 (Figure 4). On the other hand, the RMSD is very lower (0.00109) which confirm the validity of the model (Table 1).

The developed QSAR model is valid at 98%, which is in agreement with previous studies reporting a strong structure effect relationship for the proteasome 20S [29, 30, 31]. The predicted correlation of comparative molecular field analysis, and comparative molecular similarity indexes are 0.963 and 0.919, respectively (Lei et al. 2016). The difference may be due to the accuracy of the generated model [32].

Lei et al. (2016) had recently shown that 3D-QSAR models and structure-activity relationship (SAR) have an importance to develop new compounds more efficient again proteasome 20S following development of new compounds biologically more active with the importance of the radical R2 and R3 [32]. In this study, obtained results have clearly shown that the diameter and Lipinski parameters have an importance in the pIC50, leading to increase the correlation between the predictive IC50 and the pharmacokinetics values, assessed by the real IC50, reaching approximately 70% (r2: 0.89).

In this study, 71 chemicals isolated from O. europaea or F. carica species were selected to evaluate the in-silico anti-proteasome activity. These products were reported in many chemical databases, including PubChem and Zinc-Docking Database. The generated 3D model was applied on the 71 chemical to evaluate predictive structures - effects of these molecules and corresponding predictive IC50 are reported in Table 2. In Oleaeuropaea, predictive IC50 ranges from 0.008 to 6,4819E+12nM. These results showed interesting predictive IC50 and potentially good effects of O-Coumaric Acid, Cyanidin 3-Glucoside, PHydroxybenzoic Acid, Cinnamic Acid, Demethyl Oleuropein Aglycone, Ligstroside Aglycone, Oleuropein Glucoside and Hydroxybenzoic Acid on proteasome. In Ficuscarica, fewer molecules were reported in the literature and these molecules showed predicted IC50 ranging from 0.072 to 1, 0023E + 88nM. The most interesting compounds are β-bourbonene, Copaene, α- gurjunene, β-elemene, Cyanidin-3-Rutinoside, Catechin, Epicatechin, Eugenol, Linalool and Pyranoid Trans highlighting small predictive IC50 and could probably have interesting antiproteasome activities. Predictive IC50 of compounds from Oleaeuropaeaare considerably lower than those obtained with compounds from Ficuscarica. This difference could be due to the chemical structures of these molecules.

The best CoMFA models gave satisfactory results in terms of several rigorous statistical keys, such as q2 and r2, for internal and external data sets. Thus, the results obtained were used to design and for screening new molecules, which could be proven as potent inhibitors of proteasome 20S.

Activity-based and structural analyzes of the 30 reference drugs, known to be S20's inhibitors, have allowed to generate 3D-QSAR model for predictive capabilities and also to explore the mechanisms of interaction between proteasome 20S and bioactive compounds. Therefore, investigation of the chemical structure of these reference drugs together with molecules from Oleaeuropaea and Ficuscaruca exhibiting lower pIC50 will give an idea on the main structural features needed to design new potent inhibitors of proteasome 20S.The inhibitory activity of proteasome S20 predicts the proposed molecules to be quite similar based on both CoMFA and the CoMSIA models.

Structural and physiochemical characteristics of these compounds, including electron density maps, presence of OH and methyl radicals in addition to 'a radical O, are common within this group. Chemical characterization showed that the presence of an OH function (sometimes an OAc, depending on the dosage) at C-7 and C-8 electron-rich groups is essential for the associated activities.

On the other hand, the activity of some molecules, including cyanidin 3-glucoside, p--Hydroxybenzoic acid and cinnamic acid, dimethyl Oleuropein and aglycone from Oleo europaea and highlighting interesting pIC50, is mainly due to the presence of the OH, CH3 radicals and the number of rings. Moreover, in these compounds, the features of lengths were more interesting than other compounds.

Moreover, as these molecules are rather small and relatively rigid and their activities are so well defined, it seems unlikely that their observed activities can be greatly improved by modifying other functionalities. Thus, modifying the base frame in the number of rings and the distance of the molecule could be a good opportunity for improving their activities.

### Docking analysis

To confirm the theoretical results, docking analysis was used to evaluate the nature of bounds and the interactions between the plants' chemicals and the Proteasome. These analyses will give a lot of information on the affinity of these compounds to the proteasome complex. Molecular docking was done on some compounds and highlighted interesting results for oleuropein glucoside from Oleaeuropaea and, cyanidin-3-rutinoside and Epicatechin from Ficuscarica that exhibited link energies of -11.6, - 8.2 and -7.9 Kcal/mol, respectively.

**Figure 5:**
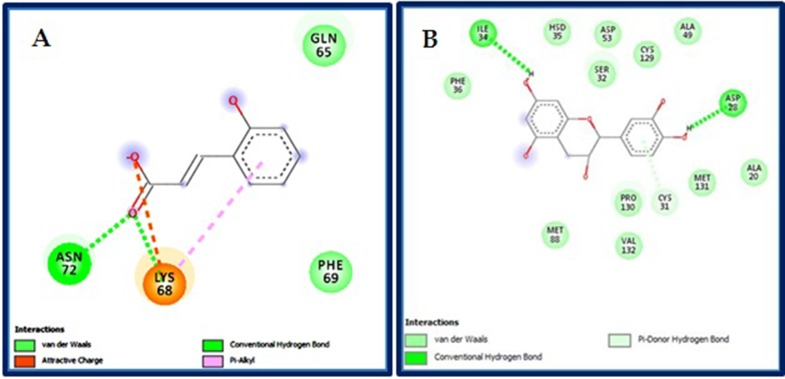
Interaction between the o-coumaric acid (A) and Catechin (B) components with the proteasome subunits I and K. View the small size of the molecules relative to the active site, we estimate that the ligand binds to several places at the active site, this leads to create non-covalent interaction leading to more effects.

Of particular interest, components with small diameters have shown high affinities to the active site of the proteasome complex. This could be due to the presence of the aromatics essentially with 5 rings and Nitrogen which increases the energy of VdW. Figure 5 presents an illustration of the main covalent bounds revealed between the o-coumaric acid, isolated from Oleaeuropaea, and Cartechin, isolated from F. carica, and active sites of proteasome 20S.

Docking analysis showed the presence of numerous non-covalent bonds, especially with the negatively charged oxygen, highlighting the importance of the radicals OH in the structures of inhibitors. These results explain the high ΔG energy and docking score obtained with many products isolated from Oleaeuropaea and Ficuscarica, making them good candidates for further investigations.

On the other hand, some bioactive compounds, isolated from O. europaea and F. carica, like Catechin, form more non-covalent bonds in active site as compared to Carfilzomib, used as a reference drug. Cartechin and Oleuropein are long molecules with large diameters, allowing them to interact and, consequently inhibit, many active residues in the active site of the proteasome.

These bioactive compounds would have the possibility to interact with the target without metabolic activation and could be a very interesting therapeutic approach to overcome the problem of resistance to available and conventional drugs.

This study is very informative and gives evidence that 2D and 3D QSAR Models and docking showed that the components of both plants having great potentialities to be S20 proteasome inhibitors. Therefore, rational tools increasingly, have a special place in the process of drug optimization and drug discovery, where QSAR 2D/3D and docking are the main tools for the optimization of process [5, 31].

### ADMET prédiction

ADMET prediction was used to evaluate pharmacokinetic characteristics of chemical compounds isolated from O. europaea and F. carica including, absorption, distribution, metabolism, excretion and toxicity. Results are summarized in Table 3. In ADMET perdition, the Plasma Protein Binding (PPB) test is used to predict the percentage of drug bound to plasma proteins. Usually, only unbound molecules are available for diffusion across cell membranes and consequently could interact with pharmacological targets. Moreover, the level of plasma protein binding of drugs influences not only their action but also their disposition and efficacy [33, 34, 35]. In this study, ADMET analyses have showed that most molecules isolated from F. carica are strongly bound to plasma proteins. However, all molecules isolated from O. europaeaare weakly bound (PPB<90%).

Blood Brain Barrier (BBB) evaluation is a crucial test in pharmacological studies in pharmaceutical sphere. In fact, CNSactive compounds must pass across BBB to interact with their respective targets. Moreover, BBB blocks most chemicals don't targeting the CNS to avoid eventual side effects [36, 37]. In this study, BBB evaluation was performed using criteria published by Ma et al. [38]. Most molecules from O.europaea have BBB values comprised between 2 and 0.1, meaning that they have a middle absorption. However, Cyanidin 3-Glucoside highlights a low absorption with BBB prediction value less than 0.1. In contrary, most of molecules isolated from F.carica are strongly absorbed, with BBB prediction values more than 0.2, excepted catechin and Epicatechin that have middle absorption (BBB value = 0.39) and cyanidin-3-rutinoside which is low absorbed (BBB value < 0.1).

Human intestinal absorption (HIA) reflects the bioavailability and absorption of drugs and is evaluated from the ratio of excretion or cumulative excretion in urine, bile and feces. This test is very crucial to identify potential drug candidate [39]. In this study, molecules from both O. europaea and F. carica were applied at pH 7.4 to predict HIA and results clearly showed that the majority of molecules from the two plants have good and moderate absorptions.

Overall, molecules from O.europaea showed good pharmacokinetic and pharmacodynamic properties and could therefore be used as proteasome targeting drugs with efficacy and safety.

## Conclusion

The development and validation of a QSAR model is of a great interest for screening chemical molecules for proteasome inhibition from plant source. Model shows that many compounds of O. europaea L and F. carica L. have potential S20 proteasome inhibition activity. Therefore, it is of interest for targeting proteasome with molecules more efficacy and safety.

## Disclosure

This research did not receive any specific grant from funding agencies in the public, commercial, or non-profit sector.

## Competing Interests

The authors declare that they have no competing interests.

## Figures and Tables

**Table 1:** 3D/ 2D QSAR model analysis.

PLS statistics	Calibration set	Training set(for all compounds)
Determination of coefficient (r2)	0.97	0.96
Root mean square error interne	0.28353	0.27
q2	0.00109	0.002

**Table 2:** Predictive toxicity of compounds from Oleo europaea and Ficuscarica

Oleaeuropaea		Ficuscarica		
Compounds	IC50 (nM)	Compounds	IC50 (nM)
o-coumaric acid	0,0085	β-bourbonene	0,0724
cyanidin 3-glucoside	0,0136	Copaene	0,1072
p--Hydroxybenzoic acid	0,0275	α-gurjunene	0,1378
cinnamic acid	0,1045	β-elemene	0,1660
Demethyl Oleuropein Aglycone	0,4824	cyanidin-3-rutinoside	0,1850
ligstrosideaglycone	12,303	Catechin	0,3740
oleuropeinglucoside	12,785	Epicatechin	0,3740
hydroxybenzoic acid	16,376	Eugenol	0,7478
Sinapic acid	4,76,431	Τ-muurolene	0,8710
Gallic acid	12,62,699	germacrene D	28,184
Vanillic acid	12,62,699	Linalool	88,450
Syringic acid	12,62,699	Pyranoid trans	1,81,134
p-Coumaric acid	12,62,699	Hexanal	10,23,293
Tyrosol	12,62,699	Furanoid (cis) linalool oxide	12,62,699
Oleuropein	12,62,699	Bergapten	71,86,212
Ferulic acid	71,02,316	quercetin-glucoside	2200,39,171
homovanillic acid	584,79,008	ketone: 3-pentanone	2884,03,150
Hydroxytyrosol	763,83,578	Angelicin	3221,06,879
Luteolin	890,02,048	Psoralen	7585,77,575
Apigenin	3497,03,517	β-cyclocitral	1.45E+09
5-caffeoylquinic acid	5154,65,971	kaempferol-rutinoside	1.60E+09
Elenolic acid	6657,32,869	Bergapten	2.29E+09
Chrysoeriol	6804,55,925	Angelicin	2.75E+09
Salidroside	1.01E+09	fumaric acid	3.42E+09
oleuropeinaglycone	2.47E+09	Astragalin	8.74E+09
Verbascoside	3.50E+09	chlorogenic acid	1.24E+11
apigenin-7-glucoside	1.48E+10	Shikimic acid	1.00E+13
Secoxyloganin	1.63E+10	Benzyl aldehyde	5.54E+25
apigenin-7-rutinoside	2.15E+10	Pyranoidcis	5.13E+32
Homoorientin	3.59E+10	Furanoid trans	1.25E+36
luteolin-4'-glucoside	4.55E+10	"apigenin-rutinoside"	1.00E+92
Ligstroside	1.38E+11		
Rutin	2.55E+11		
elenolic acid glucoside	1.00E+13		
Quercetin	1.00E+13		
Secologanin	1.00E+13		
Oleoside	1.31E+13		
Caffeic acid	8.92E+13		
luteolin-7-glucoside	2.84E+14		
Nuzhenide	6.48E+16		

**Table 3:** ADME and toxicity calculation.

ADME tests							Toxicity tests		
		BBB	Caco2	HIA	MDCK	Plasma_Protein_Binding	algae_at	Ames_test	Carcino_Mouse
Oleaeuropaea	O-Coumaric Acid	0.694635	21.1093	92.095876	75.0598	63.055072	0.10446	mutagen	negative
	Cyanidin 3-Glucoside	0.027784*	3.48966	2.916601	1.2445	27.095367	0.0301655	mutagen	positive
	P--Hydroxybenzoic Acid	0.643365	20.314	88.138567	64.8646	8.772868	0.116565	mutagen	negative
	Cinnamic Acid	1.86487	21.0342	97.8452	229.476	60.85253	0.124309	mutagen	Negative
	DemethylOleuropeinAglycone	0.0602445	20.3816	66.996823	2.06142	71.636727	0.0173954	mutagen	Negative
Ficuscarica	β-bourbonene	11.3636	23.4924	100	41.9633	94.602797	0.0171488	mutagen	Negative
	Copaene	11.1471	23.6323	100	40.0711	100	0.0169301	non-mutagen	Negative
	α-gurjunene	11.9141	22.3275	100	46.5435	100	0.0130084	non-mutagen	Negative
	β-elemene	13.4359	23.4917	100	56.8713	100	0.0170026	Mutagen	negative
	cyanidin-3-rutinoside	0.0296272	5.82222	4.879009	0.10985	70.917781	0.0037793	non-mutagen	negative
	Catechin	0.394913	0.656962	66.707957	44.3849	100	0.0287313	mutagen	negative
	Epicatechin	0.394913	2253.95	66.707957	44.3849	100	0.0287313	mutagen	negative
	Eugenol	2.25544	46.8865	96.774447	342.148	100	0.0567231	mutagen	negative
	Τ-muurolene	13.4717	23.6336	100	57.0682	100	0.0136614	mutagen	negative
BBB (Blood Brain Barrier): High absorption CNS >2.0, Middle absorption CNS 2.0-0.1, Low absorption CNS <0.1 (Ma, 2005); Caco2 High permeability >70, Middle permeability 4-70, Low permeability <4; HIA (Human Intestinal Absorbance): Well absorbed compounds 70-100%, moderately absorbed compounds 20-70%, Poorly absorbed compounds 0-20%; MDCK permeability >500, Medium Permeability 25-500, lower permeability <25; PPB (Plasma Protein Binding): Strongly Bound >90%, Weakly Bound <90%.
